# A LiDAR SLAM-Assisted Fusion Positioning Method for USVs

**DOI:** 10.3390/s23031558

**Published:** 2023-02-01

**Authors:** Wei Shen, Zhisong Yang, Chaoyu Yang, Xin Li

**Affiliations:** 1 School of Marine Science, Shanghai Ocean University, Shanghai 201306, China; 2 Shanghai Estuary Marine Surveying and Mapping Engineering Technology Research Center, Shanghai 201306, China

**Keywords:** fusion positioning, unmanned surface vessel (USV), GNSS/INS, LiDAR-SLAM

## Abstract

Confronted with unmanned surface vessel (USV) operations where GNSS signals are unavailable due to obscuration and other factors, a LiDAR SLAM-assisted fusion positioning method for USVs is proposed to combine GNSS/INS positioning with LiDAR-SLAM. When the USV works in wide-open water, the carrier phase differential GNSS/INS loosely coupled integration strategy is applied to fuse and calibrate the positioning data, and the positioning information of the USV is obtained through the coordinate conversion process. The system uses a dynamic switching strategy to enter to LiDAR-SLAM positioning when GNSS signals are not available, compensating the LiDAR data with precise angle information to ensure accurate and stable positioning. The experiments show that compared with the traditional Kalman filter and adaptive Kalman filter fusion algorithms, the positioning error is reduced by 55.4% and 43.5%. The velocity error is also limited by 78.2% and 57.9%. The standard deviation and the root mean square error are stable within 0.1 m, indicating that our method has better data stability, while the probability of positioning anomaly is effectively controlled.

## 1. Introduction

An unmanned surface vessel (USV) is an intelligent platform on water equipped with a control system, communication system, and sensor system, which minimizes navigation risk and is highly valuable for research and application in facing the complex and changing water operation environment [1,2]. USV autonomous positioning [3] and navigation [4] are the cornerstones of the realization of unmanned, commonly using sensors such as GNSS, INS, LiDAR, Doppler range meter, vision sensors, etc. The current mainstream navigation and positioning method applies multiple sensors for data acquisition and correction, fully utilizing the respective advantages of each sensor to achieve the purpose of improving navigation and positioning accuracy.

The most popular way is GNSS/INS combination navigation. GNSS has a stable signal and high positioning accuracy in the outdoor open environment, but under vegetation blockage in inland waters, the satellite cannot solve the fixed solution, resulting in signal loss of lock and data loss. Severe multipath effects [5] can also degrade positioning accuracy. The INS acquires positioning information by using self-integrating sensors over time to complete the trajectory projection, which is not subject to external signal interference and can provide high-frequency updated 3D velocity and attitude measurements in a short time, but its drift error and scale error will accumulate with long operation [6,7], and it is hardly used alone for positioning. The combination of GNSS and INS can make up for their respective shortcomings and increase the anti-interference capability of the system, which is widely used in maritime navigation. Ziebold [8] adds DVL to GNSS/IMU fusion solution, demonstrating excellent anti-jamming performance in maritime applications. Zhao [9] proposes a GNSS/INS depth coupling algorithm based on H∞ filtering technology based on robust filtering theory, with a simple structure and high state estimation accuracy. However, neither the position and velocity under loose coupling nor the pseudo-range and pseudo-range rate under tight coupling can solve the positioning problem of USVs in GNSS signal loss scenarios for long periods.

LiDAR is used for operation in areas with obvious environmental features, such as indoor or dense buildings. Tang [10] proposes a new scanning method that matches the INS of an assisted inertial navigation system with low-cost LiDAR to improve the stability of navigation information from weak GNSS signals. Kumar [11] proposes to obtain the horizontal displacement using INS and 2D-LiDAR data, the altitude change using the ground information from the second LiDAR set, and the two sets of displacement information are fused by Kalman filtering to obtain the 3D trajectory of the UAV.

To satisfy the USV positioning needs in the positioning satellite signal obscured areas, such as under a dock or bridge or in a tunnel, we propose a LiDAR SLAM-assisted unmanned surface vessel fusion positioning method ‘Navigation of USV’, abbreviated as NavUSV, which adopts a carrier-based phase-difference separation based GNSS/INS loosely coupled integration strategy to obtain the position information when working in outdoor open water. When entering an environment with weak satellite signals, the system switches to LiDAR-SLAM positioning and compensates the LiDAR data using precise angle information to ensure accurate and stable positioning.

## 2. Method Description

**Figure 1:**
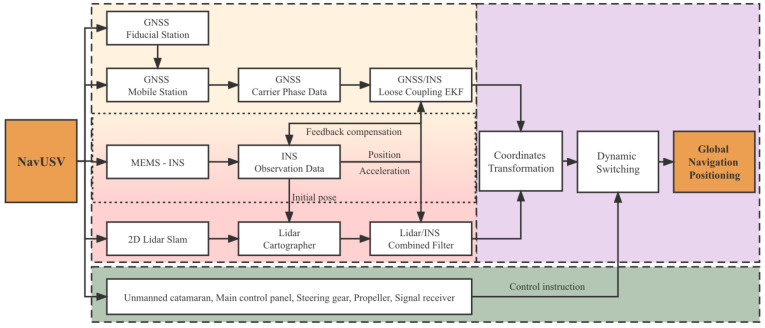
NavUSV integrated navigation method roadmap. Different colors represent different sub-sections.

A method named NavUSV is proposed for the complicated and changing positioning environment of USVs, based on the self-developed USV platform, equipped with a variety of environmental sensing and navigation and positioning sensors, using different sensor information fusion in different environments, and fully utilizing the advantages of GNSS, INS and LiDAR sensors. Thus, achieving the purpose of dynamic switching of indoor and outdoor navigation and positioning of USVs. The technical roadmap is shown in Figure 1, where the yellow box represents the GNSS/INS layer, the red box represents the LiDAR/INS layer, the purple box represents the fusion conversion layer, and the green box represents the control layer.

### 2.1. GNSS/INS Loosely-Coupled Integrated System

**Figure 2:**
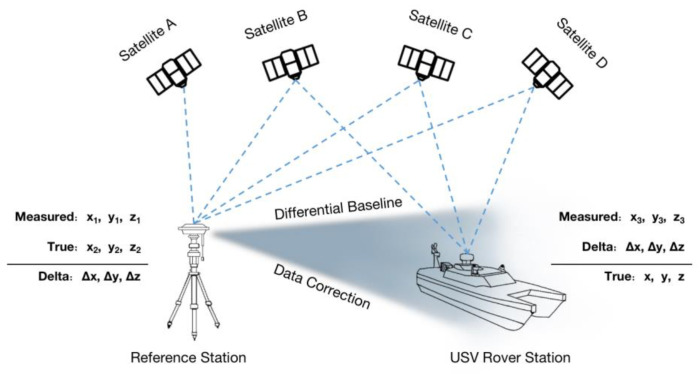
Structure of carrier phase differential GNSS system.

The carrier phase differential GNSS system includes a reference station and a mobile station. The land reference station calculates the differential data based on the known fixed latitude and longitude as well as its own measured real-time positioning, which is sent to the mobile station carried by the USV via broadcasting. The mobile station receives the differential data and its own measurement of real-time positioning data for differential processing to obtain high-precision positioning data (Figure 2).

In the loosely coupled structure, both carrier phase differential GNSS and INS work independently and provide the results of navigation parameters separately. The INS is a type of MEMS-INS, whose data errors mainly consider gyroscope and accelerometer errors. Through INS long-term static data analysis, the first-order Markov process is chosen to describe the random drift of the gyroscope, and the specific parameters are shown in Table 1. For the nonlinear robust filtering problem of the combined navigation system, NavUSV uses the extended Kalman filtering(EKF) algorithm to achieve approximate linearization by deriving the first-order Taylor expansions of the nonlinear state transfer function and the observation function [12], and discarding the higher-order terms, in which the equation of state for the current moment is estimated from the state and motion model estimated at the previous moment as:

(1) $${\hat{x}}_{k|k-1}=f\left({\hat{x}}_{k-1|k-1},\mu_{k}\right)$$

$x_{k-1}$ denotes the state estimate of the previous moment.

Handling noise using the error covariance prediction equation:

(2) $$P_{k|k-1}=F_{k}P_{k-1|k-1}F_{k}^{T}+Q_{k}$$

$Q_{k}$ is the process noise covariance matrix, and $F_{k}$ is the state transfer matrix, while $B_{k}\mu_{k}$ is the control input.

The Kalman gain is calculated to assign weights between the states predicted by the model and the states measured by the sensors:

(3) $$K_{k}=P_{k|k-1}H_{k}^{T}{\left(H_{k}P_{k|k-1}H_{k}^{T}+R_{k}\right)}^{-1}$$

$H_{k}$ is the observation model matrix, and $R_{k}$ is the observation noise covariance matrix.

Further updating of the state based on the obtained Kalman gain and measurements:

(4) $${\hat{x}}_{k|k}={\hat{x}}_{k|k-1}+K_{k}(z_{k}-H_{k}{\hat{x}}_{k|k-1})$$

$z_{k}$ is the measured value of the sensor.

In the last, the error covariance matrix is updated to be used as $P_{k-1|k-1}$ for the next iteration, and finally, the exact estimate of the state quantity of the system to be solved is obtained:

(5) $$P_{k|k}=(I-K_{k}H_{k})P_{k|k-1}$$

The system inputs the position and velocity information provided by GNSS and the position, velocity, and attitude provided by INS into the extended Kalman filter [13,14,15,16,17]. The filter builds an error model by comparing the difference between the two and outputs attitude error, position error, and velocity error to update the carrier position and velocity output by INS to obtain combined navigation results, so that the USV can output stable positioning information even when the satellite signal is obscured for a short time.

### 2.2. D LiDAR-SLAM/INS Integrated System during GNSS Outages

**Figure 3:**
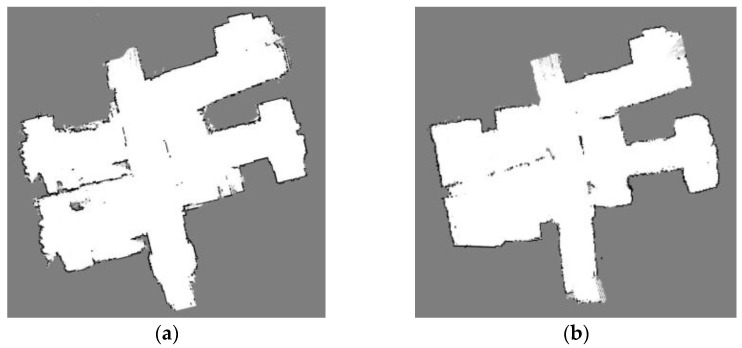
Comparison of two algorithms for simple indoor construction mapping: (**a**) Gmapping; (**b**) Cartographer.

Because the Bayesian filter-based Slam algorithm can not easily eliminate the cumulative error, such an algorithm is only suitable for interior small-area maps. At the same time, the graph optimization-based Slam can optimize the global robot poses by loopback to eliminate the cumulative error, so it has an advantage over the filter-based Slam. The Cartographer algorithm is an open-source algorithm introduced by Google in 2016 [18], which is also the best 2D-LiDAR-SLAM algorithm. It has been iterated for four versions and has excellent map-building accuracy and robustness. Figure 3 compares the effect of simple indoor building experiments using the Gmapping algorithm and the Cartographer algorithm. It can be seen that there is a small drift in the bit pose estimation during the building process of the Gmapping algorithm, which leads to a constant change in the position of obstacles in the environment, a thickening of walls, and a misaligned superposition of obstacles.

Considering that the USVs need to operate in a GNSS-challenged environment, characterized by few feature points and many linear structures or repetitive structures, we have combined MEMS-INS and 2D LiDAR-SLAM by EKF and used Cartographer to interpolate the poses together with scan matching in local SLAM to construct maps in a GNSS-challenged environment. Each combined filtering process includes a system state and observation update, specifically referring to the prediction update of the INS error state and observation update based on 2D LiDAR plane alignment results in the combined navigation system.

### 2.3. Multi-Source Information Fusion Positioning

The GNSS positioning system based on carrier phase differential is suitable for open inland rivers and lakes, etc. Combined with MEMS-INS, it can obtain high-precision positioning data, but the accuracy decreases in indoor or GNSS-challenged environments. The 2D LiDAR-SLAM-based positioning system is ineffective for outdoor positioning due to laser ranging limitations and sparse map-building feature points, whereas it is suitable for indoor relatively narrow and fixed environments. These two navigation and positioning strategies are complementary, and the proposed NavUSV method combines the two systems and dynamically switches between them in the transition region to achieve compatibility with both indoor and outdoor environment positioning requirements.

#### 2.3.1. Coordinate System Transformation

**Figure 4:**
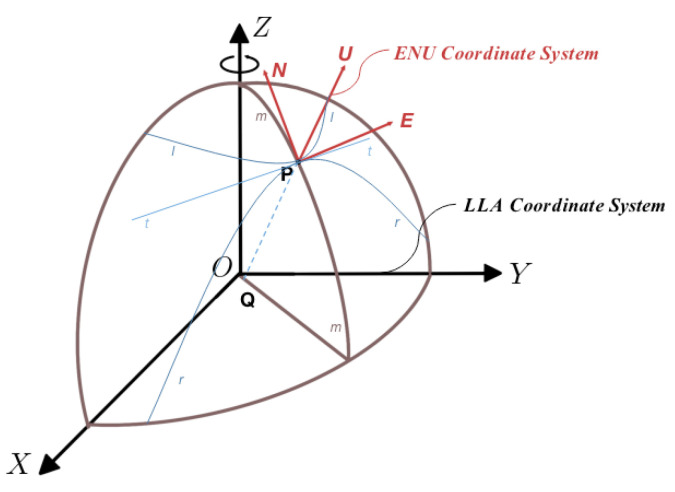
Relative position relationship between two coordinate systems.

When USVs operate in open and unobstructed waters, the GNSS system is used as the dominant navigation method. The WGS84 geodetic coordinate system gives the geodetic latitude, geodetic longitude, and geodetic elevation of a point and tells the position of the point in the Earth more intuitively, so it is also called the latitude, longitude, and elevation coordinate system (LLA). Therefore, it is necessary to convert LLA coordinate system data output from the GNSS system into the navigation coordinate system applied to USV navigation and positioning, and the east-north up coordinate system (ENU) is based on the user’s location P as the origin, with the X-axis pointing east, the Y-axis pointing north, and the Z-axis pointing to the zenith. ENU coordinate system in the geographic coordinate system is generally used as the navigation coordinate system in inertial navigation, as shown in Figure 4.

(A) Finding the rotation matrix between the WGS84 coordinate system and the Earth coordinate system

In Figure 4, let point P be a point on the rotating ellipsoid and n be the normal at point P. O−Z is the axis of symmetry of the ellipsoid, and the plane curve lPl obtained by intercepting the ellipsoid through P as the vertical plane of O−Z is called the latitudinal circle at the point P. The plane curve mPm obtained by making a plane truncated ellipsoid through point P and O−Z is called the meridian circle (meridian circle), the plane curve rPr obtained by truncating the ellipsoidal plane formed by tPt, and the normal P−U with the tangent tPt of the latitudinal circle lPl across point P is called the prime vertical circle at point P. The radius of curvature $R_{n}$ along the meridian circle mPm at point P is the radius of curvature of the meridian circle, and the radius of curvature $R_{e}$ along rPr is the radius of curvature of the prime vertical circle. The calculation formula is shown in Equations (6) and (7).

The radius of the meridian circle at height h is $R_{n}+H$, and the radius of the prime vertical circle is $R_{e}+H$. The coordinate transformation matrix between the WGS84 coordinate system and the Earth coordinate system is:

(6) $$\begin{array}{l} R_{N}=\frac{R(1-e^{2})}{{\left(1-e^{2}\sin^{2}L\right)}^{\frac{3}{2}}}\\ R_{E}=\frac{R}{{\left(1-e^{2}\sin^{2}L\right)}^{\frac{1}{2}}} \end{array}$$

(7) $$\begin{array}{l} \left[\begin{array}{l} X\\ Y\\ Z \end{array}\right]=\left[\begin{array}{l} (N+H)\cos B\cos L\\ (N+H)\cos B\sin L\\ \left[N(1-e^{2})+H\right]\sin B \end{array}\right]\\ N=\frac{a}{\sqrt{1-e^{2}\sin^{2}B}}e^{2}=\frac{a^{2}-b^{2}}{a^{2}} \end{array}$$

where R and a both denote the Earth’s equatorial radius (the long semi-axis of the ellipse) while b is the short semi-axis of the Earth’s ellipsoid, and in the WGS-84 model, a = 6,378,137 m, b = 6356752.314 m, e = 0.0818191908426, N is the radius of curvature R of the prime vertical circle, B is the latitude of point P, and L is the longitude of point P.

(B) Finding the rotation matrix of the coordinates between the Earth coordinate system and the ENU coordinate system

The rotation relationship between the Earth coordinate system and the geographic coordinate system can be represented by the direction cosine array. When the geographic coordinate system is chosen as the ENU coordinate system, its coordinate transformation matrix can be expressed as $C_{e}^{g}$, as shown in Equation (8). When the system starts, the ENU coordinate system can be established with the sensor start position as the origin, and the navigation information of the USV after UTM two-dimensional projection will be expressed as (x,y,θ).

(8) $$C_{e}^{g}=\left[\begin{array}{ccc} -\sin\lambda & -\sin L\cos\lambda & \cos L\cos\lambda \\ \cos\lambda & -\sin L\sin\lambda & \cos L\sin\lambda \\ 0 & \cos L & \sin L \end{array}\right]$$

(C) 2D LiDAR-SLAM coordinate system matching

To ensure data stability and consistency when the two systems are dynamically switched, it is necessary to use the same coordinate system to represent the data information of the USV navigating in different system combinations. Firstly, the 2D LiDAR-SLAM navigation coordinate system is rotated in the same direction as the GNSS navigation coordinate system axes, and the current attitude angle of the USV is obtained in the transition region (where both systems exist), and the difference is found to obtain the corrected rotation angle [19]. Suppose the coordinates before rotation are $\left(x_{0},y_{0}\right)$ and the coordinates after rotation are $\left(x_{1},y_{1}\right)$, the process shown in Equation (9). Assuming that the translation is (dx,dy), and the coordinate values of 2D LiDAR-SLAM navigation coordinate system after rotation and translation are $\left(x_{1},y_{1}\right)$ and $\left(x_{2},y_{2}\right)$, respectively, the specific process is as in Equation (10).

(9) $$\begin{array}{l} (x_{1},y_{1},1)=(x_{0},y_{0},1)\left[\begin{array}{ccc} \cos(\alpha ) & \sin(\alpha ) & 0\\ -\sin(\alpha ) & \cos(\alpha ) & 0\\ 0 & 0 & 1 \end{array}\right] \end{array}$$

(10) $$(x_{2},y_{2},1)=(x_{L1},y_{L1},1)\left[\begin{array}{ccc} 1 & 0 & 0\\ 0 & 1 & 0\\ d_{x} & d_{y} & 1 \end{array}\right]$$

After unifying the coordinate systems of the two navigation systems, the navigation information $\left(x_{2},y_{2},\theta \right)$ of the 2D LiDAR-SLAM coordinate system will be obtained. The USV position information before the completion of the dynamic switching of the navigation system will also be transmitted and used as the initial position of the USV of the navigation system after the dynamic switching to improve the stability and consistency of the USV navigation and positioning data.

#### 2.3.2. Dynamic Sensor Switching Framework

**Figure 5:**
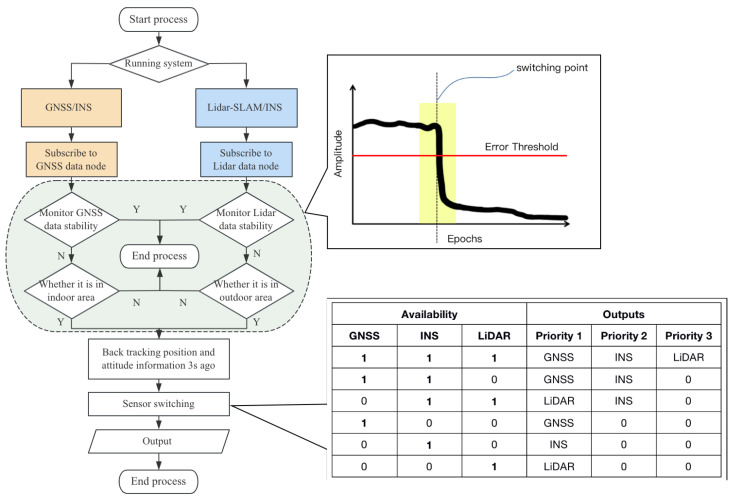
Dynamic sensor switching process of NavUSV.

**Figure 6:**
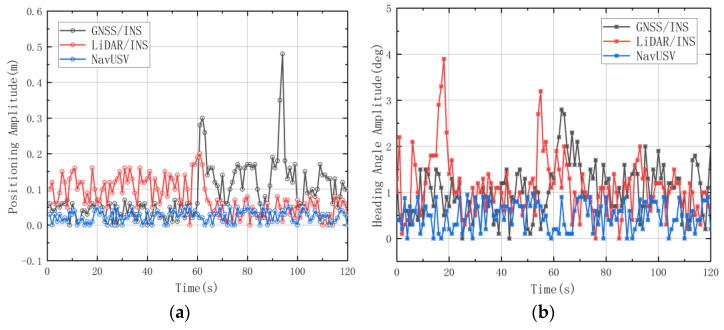
Data amplitude for dynamic switching of sensors: (**a**) Positioning data amplitude before and after switching; (**b**) Heading angle amplitude before and after switching.

(1) To achieve continuous and stable navigation of the USV navigation system, real-time monitoring of the output positioning information and attitude data is required when passing between the scenarios to which each of the two combined navigation methods is adapted. The monitoring content mainly includes the reliability of the navigation information and the environmental monitoring status information of each of the two combined navigation modes. The monitoring method is to subscribe to the position data and environmental status data of the GNSS and LiDAR positioning system and then analyze the reliability, according to the current position of the USV, to determine whether it is necessary to switch to another navigation mode, as shown in Figure 5. 

(2) Each observable sensor in the loosely coupled architecture is relatively independent of each other, so the sensor switching strategy can be described as monitoring the performance of the positioning system in terms of the total system error (TSE) confidence, alerting the sensor node when the performance metric of the working sensor exceeds a specified threshold [20,21], and the USV control system decision layer responds promptly and predicts the availability of the current positioning system to decide whether to switch from the current sensor to a set alternative positioning information source.

(3) When the USV drives into the GNSS-challenged environment, the GNSS signal generates a lot of fluctuations and positioning anomalies. The LiDAR also changes from being heavily affected by water reflections in open water to receiving obvious obstacle reflection signals. At this point, the TSE exceeds the preset threshold. The GNSS node issues an alarm, and the USV control layer determines the need to switch to the LiDAR-SLAM positioning information source. It retraces the position information of the USV in the combined GNSS/INS system 3s ago, inputs and adopts it as the initial position of the combined LiDAR system, and the coordinate system transformation is carried out.

(4) When the USV enters a GNSS signal stabilization environment, the GNSS signal availability improves significantly and smooths out. At this time, the performance of the LiDAR positioning system falls out of the threshold, the availability decreases, the USV control layer judges that the node status of the combined GNSS/INS system returns to normal, and the USV’s positional information before 3s in the LiDAR-SLAM system is inputted into the coordinate system of the combined GNSS/INS system accordingly. According to the experimental test, the error of positioning data before and after switching between the two positioning systems is less than 5cm, and the fluctuation of the heading angle is less than 1°, as shown in Figure 6.

## 3. NavUSV-Based USV Positioning Experiments

### 3.1. USV Hardware Platform

**Figure 7:**
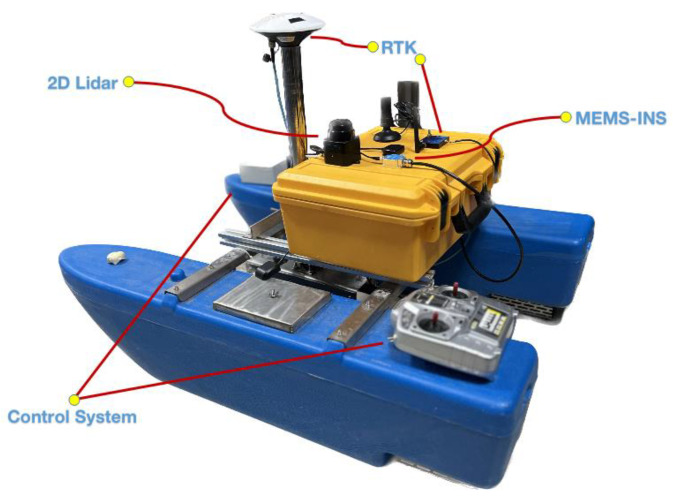
Unmanned craft platform and different sensors.

This research is based on the self-developed multi-module intelligent catamaran unmanned boat platform, which contains a control module, a navigation and positioning module, and an operation module. The hull contains a homemade control box with multiple ports containing GNSS signal input port, 485 electronic compass communication, left and right motors, and power supply, which can realize the functions of external manual control, internal autonomous cruise, and navigation security data acquisition and processing. The USV is equipped with carrier phase differential GNSS/INS and 2D LiDAR-SLAM navigation and positioning systems, both rigidly connected to the USV operating platform, as shown in Figure 7.

The GNSS positioning system adopts the WTRTK-WL module, the reference station is erected on the shore of the operating waters of the USVs, and the mobile station is erected on the working navigation platform of the USVs. The INS module adopts WTGAHRS3 3D motion attitude measurement system based on MEMS technology, which contains a 3-axis gyroscope, 3-axis accelerometer, and other motion sensors. The parameters are shown in Table 1. The 2D LiDAR-SLAM positioning system uses the SLAMTEC Mapper LIDAR sensor, which supports more than 10 times per second map data fusion and up to 100,000 square meters of map data mapping. The LiDAR performs 9200 ranging actions per second with a maximum ranging distance of 40 m, and detailed technical specifications are shown in Table 2.

### 3.2. Sailing Test

To verify the reliability and accuracy of the USV positioning system, 2 validation experiments were conducted on the campus of Shanghai Ocean University. In this research, the sample rates of IMU and GNSS are 200 Hz and 10 Hz, respectively, and the parameters of LiDAR can be obtained from Table 2. The initial state vector and the associated covariance are set as x**_0 | 0_** = [0_1×9_, 0.1 deg/s, 0.1 deg/s, 0.1 deg/s, 10 mg, 10 mg, 10 mg, 0.001 deg/s, 0.001 deg/s, 0.001 deg/s, 0.1 mg, 0.1 mg, 0.1 mg], and P**_0 | 0_** = diag([0.005 rad, 0.015 rad, 0.005 rad, 0.1 m/s, 0.1 m/s, 0.1 m/s, 5 m, 10 m, 5 m, 0.1 deg/s, 0.1 deg/s, 0.1 deg/s, 10 mg, 10 mg, 10 mg, 0.001 deg/s, 0.001 deg/s, 0.001 deg/s, 0.1 mg, 0.1 mg, 0.1 mg])^2^. The base station and antenna we used in the differential GNSS system is Southern Satellite Navigation’s Galaxy 1 RTK, with a sampling rate of 1Hz to 20Hz. We use a patch antenna from ublox to collect data as a set of reference datasets. The proposed algorithm and existing algorithms are all carried out by ROS and Matlab 2016 on a computer with an Intel core i5-4200H CPU at 2.80 GHz, 8 GB memory, and Windows 10 operating system.

Experiment I

**Figure 8:**
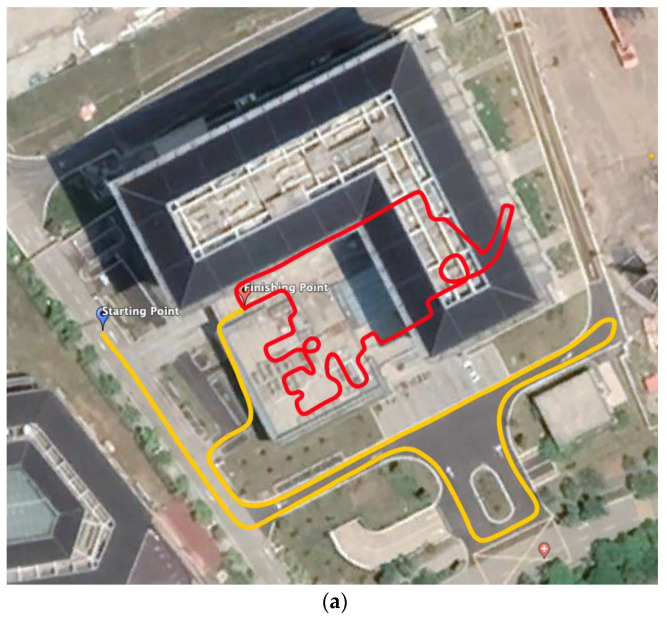

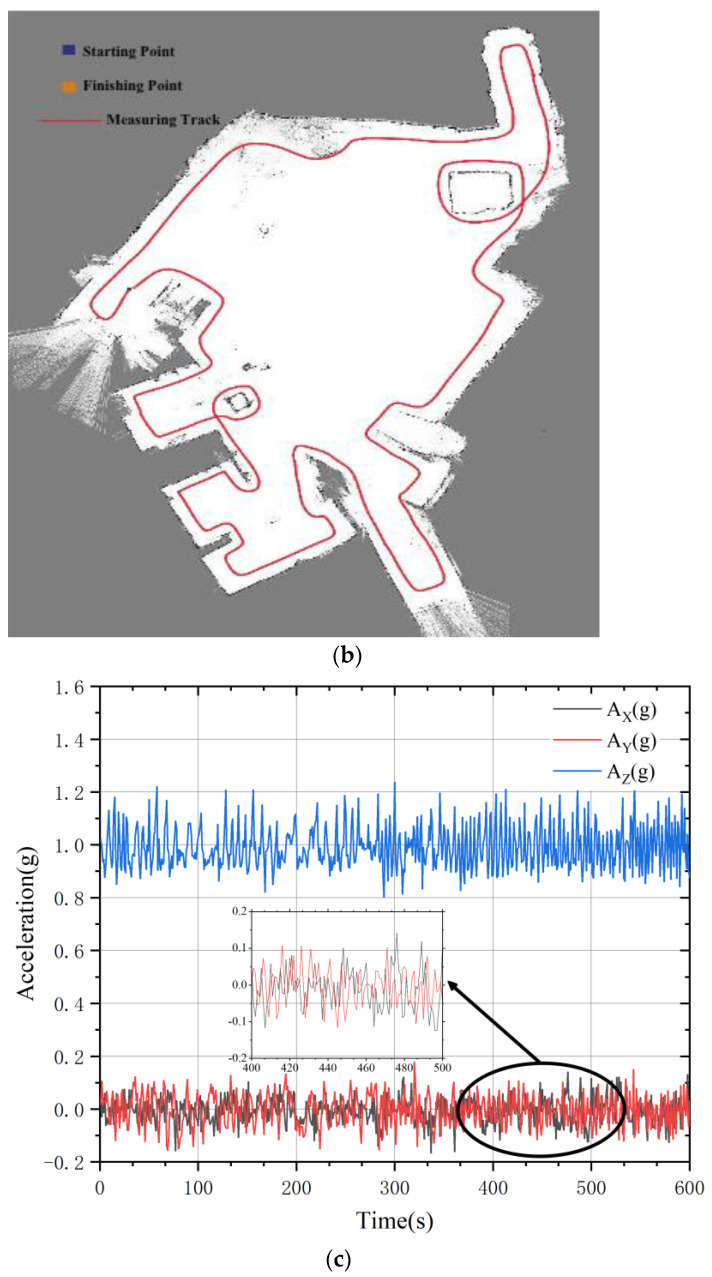
Experiment I navigation test: (**a**) Outdoor navigation route, red represents indoor track, yellow represents outdoor track; (**b**) Indoor synchronize mapping; (**c**) System acceleration after fusion.

The system feasibility experiment was carried out in a building on campus, and the test path is shown in Figure 8a. The yellow line indicates the outdoor path using the combined GNSS/INS positioning mode, and the red line indicates the indoor path using the combined LiDAR-SLAM/INS positioning. The total test time was 600 s, including 25 s for calibration and 200 s for outdoor GNSS/INS loosely coupled navigation positioning. The indoor area was entered at the 250th second with 2D LiDAR-SLAM/INS combined positioning and simultaneous indoor mapping, which took 350 s, as shown in Figure 8b.

Figure 8c shows the acceleration data obtained from INS. Figure 7c shows the acceleration data obtained from INS. A_X_, A_Y,_ and A_Z_ are the longitudinal acceleration, lateral acceleration, and vertical acceleration in the navigation coordinate system, respectively. It can be seen that through sensor information fusion and feedback correction, the white noise and zero offset generated by the system have been effectively removed, and the acceleration variation is stabilized.

Experiment II

**Figure 9:**
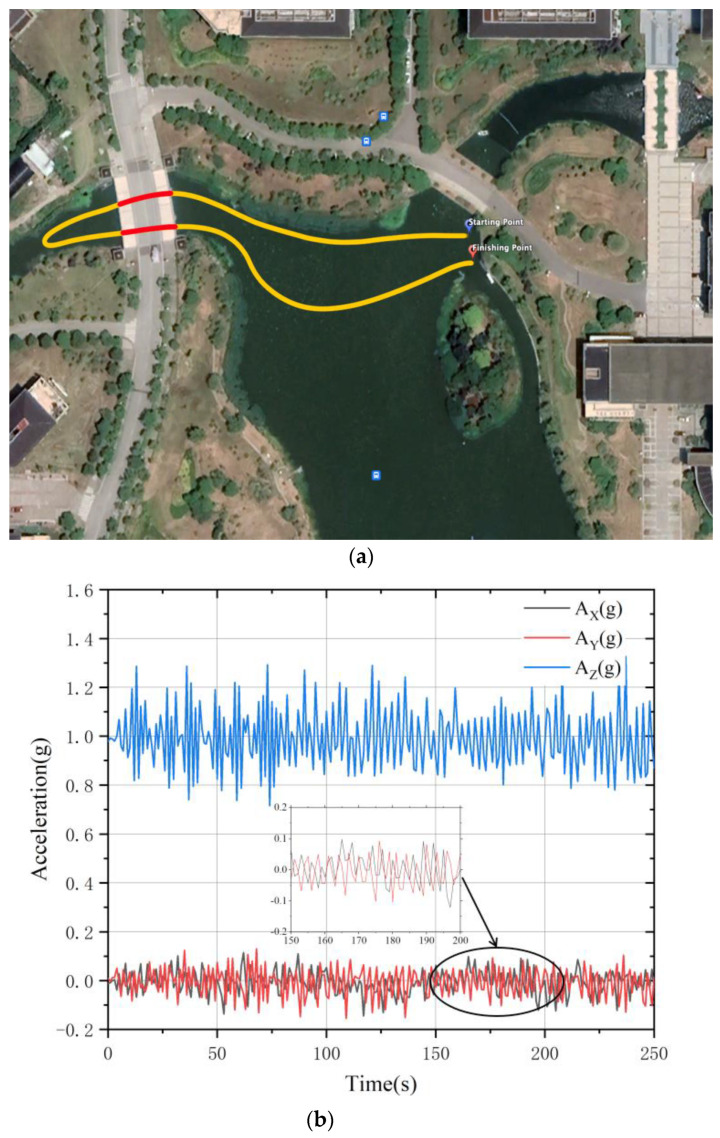
Experiment II navigation test: (**a**) navigation route, red represents track in GNSS-challenged environment, yellow represents outdoor track; (**b**) System acceleration after fusion.

Further USV crossing tests were conducted on campus lake, where GNSS signals were severely obscured under the bridge. The navigation path and the acceleration performance of the INS are shown in Figure 9a,b with the yellow line indicating the path in open water using the combined GNSS/INS positioning mode and the red line indicating the path under the bridge using the combined LiDAR/INS positioning mode.

As can be seen in Figure 9b, the three-axis deviations of A_x_, A_y_, and A_z_ are all stable within 0.2 m/s^2^, with relatively stable and controllable variations.

## 4. Results and Analysis

**Figure 10:**
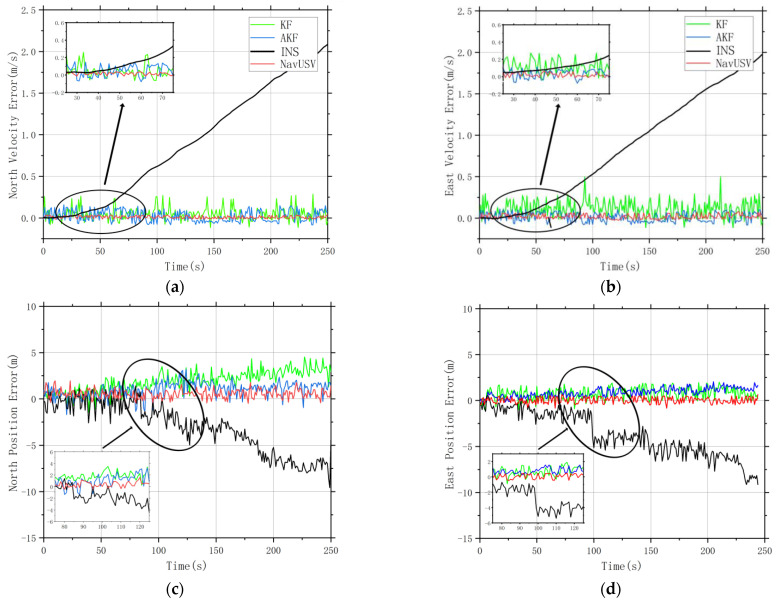
Position error and velocity error under different fusion algorithms: (**a**) North Velocity Error; (**b**) East Velocity Error; (**c**) North Position Error; (**d**) East Position Error.

We conducted the performance analysis and accuracy verification of the USVs positioning data collected in Experiment II. In the global combined GNSS/INS and LiDAR/INS positioning system, we compared the position error and velocity error of the sensor information under different fusion algorithms. It can be seen from Figure 10 that the position data under pure INS positioning produce cumulative drift error from 50 s, and the positioning data corrected by the Kalman filter and adaptive Kalman filter fusion basically maintain a stable level. The error increases when the system is run cumulatively for a longer time, while NavUSV uses an extended Kalman filter algorithm, and the error is stabilized at a constant level even after a long period of operation.

**Figure 11:**
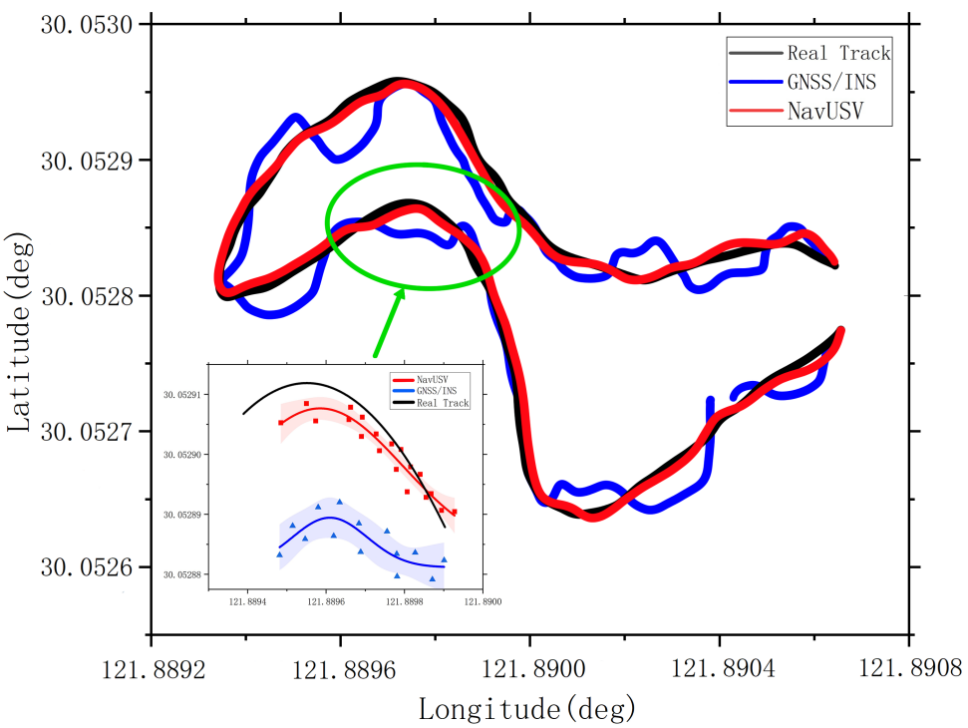
Comparison of tracks by using INS and NavUSV integrated navigation.

Figure 11 compares the navigation trajectory of the USV using the NavUSV combined navigation system with that using a single sensor (e.g., INS). It can be found that the single INS positioning without error correction has a large positioning deviation, focusing on zooming in on the trajectory offset generated by the dynamic switching of the positioning system before and after the USV passes through the bridge cave, extracting the positioning points in this region, fitting a nonlinear curve using Gaussian function, and adding a 95% confidence interval. The fitted positioning curves show that the pure GPS positioning has been significantly deviated due to environmental influence and other factors, while the curve fitted by the method still maintains a high degree of agreement with the real trajectory. Therefore, the stability and accuracy of the fused positioning of USVs under the NavUSV system are verified.

The results of this experiment are further analyzed quantitatively by comparing the root mean square error (RMSE) and the maximum error (MAX) of the information fusion results of the Kalman filter (KF), the adaptive Kalman filter (AKF), and the EKF algorithm in NavUSV to verify the superiority of NavUSV in the navigation and positioning of USVs. The root mean square error (RMSE) is the square root of the ratio of the square of the deviation of the predicted value from the true value to the number of samples m. By comparing the RMSE, we can find the difference in the error size of different methods. Usually, the RMSE of stable algorithms is stable and tends to be close to 0, and the standard deviation (SD) reflects the dispersion of each error indicator. The comparison results are shown in Table 3.

In comparison, the RMSE of the NavUSV method proposed in this research is significantly better than the other two fusion algorithms, and the positioning error is reduced by 55.4% and 43.5% compared with the KF and AKF algorithms, respectively, while the velocity error is reduced by 78.2% and 57.9%. The 95% confidence interval can be described as the point estimate of the overall proportion and the marginal error ε describing the precision of the estimated quantity; the smaller the ε, the more precise the estimate. The results show that the localization accuracy is higher using the NavUSV method within a given 95% confidence level. The standard deviation and the root mean square error are stable within 0.1m, which shows that NavUSV has better data stability than the other two fusion algorithms. In addition, the peak value of MAX for each index error of NavUSV is also smaller, indicating that the probability of outliers in the positioning process is reduced. In summary, the superiority and accuracy of the NavUSV system are guaranteed.

## 5. Conclusions

The use of single-sensor positioning in the water operation scenario of USVs cannot meet the required accuracy requirements. The mainstream approach is to adopt multiple sensors for information fusion and data feedback correction, fully utilizing the respective advantages of each sensor to achieve the purpose of improving navigation and positioning accuracy [22,23]. The accuracy of GNSS positioning is strongly correlated with signal strength and is sensitive to occlusion [24]. In some specific scenarios, it is necessary to bring in other sensors to achieve precise positioning without GNSS signals [25]. The specific work of this study is as follows:

(1) A LiDAR SLAM-assisted multi-source information fusion positioning method for USVs is proposed in this study, which innovatively integrates combined GNSS/INS positioning with a 2D LiDAR-SLAM positioning method on a USV platform. A carrier-phase differential GNSS/INS loosely coupled integration strategy is applied when the USV works in open water. Fusion correction of positioning data is performed using extended Kalman filtering, as well as coordinate conversion to obtain USV positioning information.

(2) When the USV enters the environment with a weak satellite signal, the working sensor’s performance indicator exceeds the specified threshold and raises an alarm. The USV control system responds quickly by switching from the current sensor to the set LiDAR-SLAM positioning information source. The outdoor location data with the local coordinate system as the origin are used as the initial location for building the indoor operation environment, combines the INS position data with the 2D LiDAR-SLAM method, and performs data fusion via the Cartographer algorithm to get the accurate angle information and compensates the LiDAR data to improve the stability of positioning.

(3) The feasibility of the NavUSV system is verified through experiments. Compared with other positioning methods, this research considers the positioning requirements of the USV in real operational scenarios. It utilizes an organic combination of two integrated systems so that the USV can still obtain high-precision positioning information when working in complex scenarios. In terms of accuracy, compared with the most widely used KF and AKF fusion algorithms, NavUSV reduces the positioning error by 55.4% and 43.5%, respectively, while the velocity error is reduced by 78.2% and 57.9%. The standard deviation and the root mean square error are stable within 0.1 m, showing that NavUSV has better data stability than the other two fusion algorithms. The possibility of positioning anomaly is also controlled.

(4) At present, there are two main methods of weakening multipath errors: hardware processing and post-processing. The hardware processing method is solved by new antennas and modified signal tracking loops. The method of using SNR to weaken the multipath effect is one of the post-processing methods, which analyzes the signal-to-noise ratio SNR of GPS signals and uses the frequency characteristics to weaken the multipath effect. Our solution is to use complementary GNSS/INS loosely coupled features to perform adaptive estimation of measurement noise. Then choose a properly shaped and well-shielded antenna, such as a choke antenna, and use multiple GNSS receivers in close proximity for positioning. The multipath parameters can be corrected using spatio-temporal processing to reduce the effect of multipath. 

(5) Subsequent research will try to introduce sensor data such as 3D LiDAR-SLAM and Doppler velocity log (DVL) to calculate the position and attitude of USVs with higher accuracy [26,27]. In addition, the efficacy of sensor switching depends on the fidelity of the models and databases used to predict system performance and the sensitivity of the decision thresholds. In future work, we will investigate the relevant sensor/system performance models and decision rules to set trust priorities for each sensor part and to switch the working sensors adaptively to achieve seamless and robust operation of USVs while ensuring intermittent sensor accuracy and availability. The influence of USV kinematics will be incorporated into the model building, considering the ship’s own inertia and environmental hydrodynamic, wind, and water resistance to better optimize the information fusion algorithm and improve the positioning performance of the USVs.

## Figures and Tables

**Table 1:** INS Parameters.

INS Parameter	Parameter Value
Update Rate	200 Hz
Gyro Range	±2000°/s
Gyro RMS Noise	0.05°/s
Accelerometer Range	±16 g
Accelerometer RMS Noise	0.75~1 mg

**Table 2:** SLAMTEC Mapper technical norm.

Index Content	Value
Measuring Distance	40 m
Sampling Frequency	9200 Hz
Mapping Resolution	0.05 m
Maximum Inclination Angle	±3°

**Table 3:** Statistical analysis of information fusion with KF, AKF, and NavUSV.

Algorithm	Target	East Position	North Position	East Velocity	North Velocity	Horizontal Error
		/m	/m	/(m/s)	/(m/s)	/m
KF	RMSE	1.0223	1.2892	0.1473	0.0886	1.4362
	SD	1.1678	2.4039	0.7210	0.7011	1.6373
	MAX	1.9876	3.1923	0.4969	0.2877	3.4387
	ε	-	-	-	-	0.1243
AKF	RMSE	0.9915	0.9203	0.0569	0.0727	1.2249
	SD	0.8543	1.6094	0.2534	0.4314	1.0761
	MAX	2.0592	4.5312	0.1098	0.1599	3.7086
	ε	-	-	-	-	0.0968
NavUSV	RMSE	0.3380	0.7241	0.0331	0.0188	0.9462
	SD	0.5819	0.3067	0.1738	0.1374	0.9019
	MAX	0.6584	1.9537	0.0821	0.0359	2.2197
	ε	-	-	-	-	0.0822

## Data Availability

No new data were created or analyzed in this study. Data sharing does not apply to this article.
